# Is trabecular bone score associated with multiple fragility fractures? Evidence from a large observational cohort study

**DOI:** 10.1093/rheumatology/keag551

**Published:** 2026-10-01

**Authors:** Hamzah Amin, Marwan Bukhari

**Affiliations:** Lancaster University Medical School, Faculty of Health and Medicine, Lancaster University, Lancaster, UK; Department of Rheumatology, University Hospitals of Morecambe Bay NHS Foundation Trust, Lancaster, UK; Lancaster University Medical School, Faculty of Health and Medicine, Lancaster University, Lancaster, UK; Department of Rheumatology, University Hospitals of Morecambe Bay NHS Foundation Trust, Lancaster, UK

**Keywords:** TBS, fragility fractures, multiple fractures, fracture cascade, bone quality

## Abstract

**Objectives:**

Trabecular bone score (TBS) is a measure of bone microarchitecture associated with hip and major osteoporotic fractures. However, TBS has not been evaluated in UK-specific cohorts, and little is known about its association with multiple fragility fracture phenotypes; this study aimed to investigate these associations.

**Methods:**

TBS was available in a subset of patients referred for dual-energy X-ray absorptiometry assessment in northwest England between June 2004 and May 2025. Fragility fracture phenotypes were defined as a single hip fracture (SHF), single non-hip fracture (SNHF), multiple non-hip fractures (MNHF) and multiple fractures including at least one hip fracture (MFH). These categories served as the dependent variable in a multinomial logistic regression model. Ordinal logistic regression models assessed associations with ordered hip and non-hip fracture categories. All models were adjusted for FRAX variables and the mean TBS, with multiple comparisons accounted for using Bonferroni correction.

**Results:**

A total of 2782 patients were included, with a mean age of 66.84 years (SD 12.31), mean BMI of 27.31 kg/m^2^ (SD 5.91), and 2146 (77.2%) were female. Lower TBS was associated with SNHF (OR 1.37, 95% CI 1.28–1.48), MNHF (OR 1.32, 95% CI 1.27–1.36) and MFH (OR 1.68, 95% CI 1.16–2.43). In ordinal models, lower TBS was associated with increasing fracture burden within the non-hip fracture groups (OR 1.31, 95% CI 1.16–1.47) but was not associated with SHF or the hip fracture groups.

**Conclusion:**

TBS is associated with multiple fragility fracture phenotypes and greater fracture burden within non-hip fracture categories.

*Rheumatology* key messages

- Trabecular bone score (TBS) is associated with single non-hip, multiple non-hip and multiple fractures including at least one hip.
- TBS was associated with more severe non-hip fracture phenotypes but not more severe hip fracture phenotypes.
- TBS may have utility in identifying patients with a higher burden of fragility fractures.

## Introduction

Osteoporosis is a systemic skeletal disorder characterized by reduced bone mass and microarchitectural deterioration, resulting in increased bone fragility and susceptibility to low-impact fragility fractures [1, 2]. Fragility fractures, occurring from minimal trauma such as a fall from standing height, represent a major public health concern, contributing to significant morbidity, mortality and healthcare costs [3, 4]. Hip fragility fractures are an especially problematic fragility fracture carrying a one-year mortality of up to 30% [4]. Given that osteoporosis is largely asymptomatic, clinical management focuses on identifying high-risk individuals before fractures occur using fracture risk assessment tools [5]. Commonly used tools include FRAX and QFracture, which integrate demographic, clinical and bone mineral density (BMD) measured from dual-energy X-ray absorptiometry (DXA) scans [6].

Recent advances have expanded the definition of osteoporosis to include not only reduced BMD but also compromised bone microarchitecture, captured by the trabecular bone score (TBS) [7]. TBS is a grey-level texture measurement derived from routine lumbar spine DXA images that quantifies bone microarchitecture [8]. Commonly used thresholds classify TBS values of 1.35 or higher as normal, 1.20–1.35 as partially degraded and 1.20 or lower as degraded bone [9]. TBS is valuable in the assessment of bone health as it provides additional insight into bone quality that BMD alone cannot capture, thus improving fracture risk prediction [8].

TBS has been shown to predict fragility fractures independently of BMD and FRAX. Meta-analyses report that for each one standard deviation decrease in the TBS this confers a 44% increased risk of major osteoporotic fractures [10] and around a 20% increase in hip fracture risk [11]. Clinically, TBS has been integrated into FRAX via adjustment algorithms where inputting TBS modifies 10-year fracture probabilities and often reclassifies patient risk, particularly among those with osteopenia or borderline FRAX scores [12]. Population-based studies have also shown that TBS-adjusted FRAX values changes the number of patients meeting pharmacologic intervention criteria, offering a more holistic assessment of skeletal fragility beyond BMD and traditional FRAX inputs [13].

Despite robust international evidence linking TBS to fracture risk [10], important gaps remain. Firstly, to our knowledge, no UK-based cohorts were included in previous meta-analyses, and no studies have specifically evaluated TBS as a predictor of fragility fracture within a UK clinical cohort [10]. Although TBS is an established fracture predictor, its generalizability across healthcare systems and referral populations cannot be assumed, and validation in routine UK practice is needed. Moreover, while there are no published data on TBS uptake in UK DXA services, it is likely limited in practice, given constrained scanner capacity and the additional cost of licensed software, highlighting the need for evidence to support wider adoption. Secondly, the role of TBS in predicting multiple fragility fractures and more complex fracture phenotypes is also underexplored. Multiple fragility fractures occur in up to 32% of patients within one year of an initial fragility fracture, contributing substantially to morbidity, mortality and healthcare burden [14]. TBS may be valuable in identifying patients at risk of recurrent fractures, as it captures skeletal microarchitectural deterioration beyond areal BMD [15]. These gaps in the literature form the rationale for the present study.

## Aims

The aim of this study is to investigate the relationship between TBS and varying fracture phenotypes including single and multiple fragility fractures in a large UK clinical observational cohort.

## Methods

### Data collection

Patients were referred to our regional DXA scanner in northwest England between June 2004 and May 2025. At the time of each scan, patients underwent a standardized consultation to collect FRAX risk factors, including age, sex, current or previous glucocorticoid use (prednisolone equivalent ≥5 mg daily for ≥3 months), prior fragility fracture, family history of hip fracture, current smoking status, alcohol consumption (≥3 units daily) and rheumatoid arthritis. Height and weight were recorded at the consultation and used to calculate body mass index (BMI). Information was also available on patient-reported fragility fracture sites, including hip, vertebrae, wrist and humerus.

### DXA scan protocol and trabecular bone score measurement

From 2004 until November 2019, scans were performed using a GE Lunar Prodigy system. The scanner was upgraded to an iDXA system in December 2019, and both systems were cross-calibrated to ensure methodological consistency. Weekly quality control was conducted using scan phantoms. Patients underwent bilateral femoral and lumbar spine scans following ISCD protocol, which calculated local bone mineral density (BMD) at both regions [16]. For the femoral region, BMD was calculated at the femoral neck, Ward’s triangle, femoral head, trochanters and parts of the femoral shaft.

TBS V4 (Medimaps, Geneva, Switzerland) [17] was run retrospectively on a subset of scans stored on the scanner software (enCORE). TBS V4 values were obtained for each lumbar vertebra, and a mean TBS V4 value was calculated for each patient. The mean TBS value was the key TBS parameter used in our statistical models.

### Statistical analysis

#### Primary analysis

All patients with available TBS data were included. Fracture phenotypes were first defined as: no fracture, single hip fracture, single non-hip fracture, multiple non-hip fractures and multiple fractures including at least one hip fracture. Baseline FRAX variables, BMD and mean TBS were compared across groups using a one-way ANOVA test for continuous variables and Pearson’s chi-squared test for categorical variables. Next, fracture phenotypes were compared with TBS thresholds [normal (≥1.35), partially degraded (1.20–1.34) and degraded (≤1.20)] with differences in category distribution evaluated using the Pearson’s chi-squared test. The final exploratory analysis compared the mean TBS values across the different fracture phenotypes using a one-way ANOVA test with a post-hoc Tukey’s HSD (Honestly Significant Difference) test. Statistical significance was set as *P* < 0.05.

Multinomial logistic regression was used to model the association between TBS and fracture phenotypes, with the no-fracture group serving as the reference category. The dependent variable comprised the five fracture phenotypes, while covariates included age at scan, sex, BMI, current glucocorticoid use, rheumatoid arthritis, excess alcohol consumption (≥3 units/day), current smoking status, family history of fracture, left total femoral T-score (i.e. lowest T-score from the entire proximal femoral region as defined above) and the mean TBS. Associations were expressed as the odds ratio (ORs) per 0.1-unit decrease in TBS, where lower TBS indicates worse bone microarchitecture and represented with a higher odds ratio in our model. To account for multiple testing across fracture phenotype comparisons, *P*-values were adjusted using a Bonferroni correction.

#### Secondary analysis

Secondary analyses were performed to investigate whether TBS demonstrated a graded association across ordered fracture phenotypes. Two ordinal groups were defined representing increasingly worse fracture phenotypes. Ordered hip fracture categories were defined as no fracture, single hip fracture and multiple fractures including at least one hip fracture. Ordered non-hip fracture categories were defined as no fracture, single non-hip fracture and multiple non-hip fractures. First a Cochran-Armitage trend test was used to evaluate whether the proportion of participants with degraded TBS (TBS ≤1.20) changed across severity categories. Finally, ordinal logistic regression models were subsequently fit for each group (hip and non-hip), as separate models, using the same covariates as the primary analysis, with ORs reported per 0.1-unit decrease in TBS. As an example, an OR >1 indicates that lower TBS is associated with increased odds of being classified in a higher fracture severity category. Bonferroni correction was used when evaluating the two ordered groups. Model diagnostics including the assessment of collinearity using the variance inflation factors was performed and confirmed to be adequate prior to reporting any models in this study.

### Ethics statement

Ethical approval for pseudonymized data extraction in the absence of informed consent was granted by the Northwest Preston NHS Research Ethics Committee (project number 21/NW/0309). The need for informed consent was waived by the ethics committee. The dataset did not include any minors.

## Results

### Primary analysis

#### Baseline demographics

Patients with more severe fracture phenotypes were generally older, had lower BMI, lower mean TBS and lower left femur T-scores (all *P < *0.001). Female sex was more prevalent among patients with any of the five fracture phenotypes, particularly those reporting multiple fractures, while a family history of fracture was most common in individuals with multiple non-hip fractures. In contrast, smoking status, excess alcohol consumption and rheumatoid arthritis did not differ significantly between fracture phenotypes. These results can be seen in Table 1.

**Table 1:** Baseline demographics compared between the different defined fracture phenotypes.

Variable	Overall (*n* = 2782, 100%)	No fracture (*n* = 1517, 54.53%)	Single hip fracture (*n* = 156, 5.61%)	Single non-hip fracture (*n* = 763, 27.43%)	Multiple non-hip fractures (*n* = 215, 7.73%)	Multiple fractures incl. hip (*n* = 131, 4.71%)	*P*-Value
Age at scan (years)	66.84 (12.31)	65.66 (13.06)	70.57 (11.63)	67.93 (11.40)	66.03 (9.73)	71.08 (10.76)	<0.001
BMI (kg/m^2^)	27.31 (5.91)	27.60 (6.09)	26.30 (5.41)	27.34 (5.87)	26.98 (4.98)	25.43 (5.66)	<0.001
Mean TBS (v4)	1.27 (0.10)	1.29 (0.09)	1.26 (0.09)	1.26 (0.10)	1.25 (0.09)	1.22 (0.09)	<0.001
Left femoral total T-score	−1.20 (1.22)	−0.98 (1.21)	−1.99 (1.18)	−1.36 (1.20)	−1.55 (1.03)	−2.26 (0.90)	<0.001
Female sex, *n* (%)	2146 (77.14%)	1097 (72.31%)	114 (73.08%)	636 (83.36%)	191 (88.84%)	108 (82.44%)	<0.001
Current smoker, *n* (%)	180 (6.47%)	107 (7.05%)	15 (9.62%)	38 (4.98%)	11 (5.12%)	9 (6.87%)	0.14
Excess alcohol use, *n* (%)	247 (8.88%)	135 (8.90%)	10 (6.41%)	76 (9.96%)	18 (8.37%)	8 (6.11%)	0.46
Current glucocorticoid use, *n* (%)	388 (13.95%)	277 (18.26%)	11 (7.05%)	65 (8.52%)	23 (10.70%)	12 (9.16%)	<0.001
Rheumatoid arthritis, *n* (%)	240 (8.63%)	148 (9.76%)	13 (8.33%)	52 (6.82%)	14 (6.51%)	13 (9.92%)	0.13
Family history of fracture, *n* (%)	590 (21.21%)	305 (20.11%)	22 (14.10%)	173 (22.67%)	63 (29.30%)	27 (20.61%)	0.00

Values: mean (SD) for continuous variables; *n* (%) for categorical variables. Continuous variables compared across all five fracture phenotype groups using one-way ANOVA; categorical variables compared using Pearson’s chi-squared test. Bold *P*-values indicate *P* < 0.05. TBS: trabecular bone score.

#### Distribution and average TBS values across fracture phenotypes

When examining the distribution of patients within TBS categories and across fracture phenotypes, the proportion of patients classified as having degraded trabecular microarchitecture generally increased amongst individuals with more severe fracture phenotypes (not formally tested in this table). However, across all fracture groups, most patients remained within the partially degraded TBS category. For example, amongst patients with multiple non-hip fractures, 14.9% were classified as having normal TBS, 56.3% as partially degraded and 28.8% as degraded. Patients with multiple fractures including at least one hip fracture demonstrated the highest proportion of degraded TBS within their respective phenotype group (41.2%), alongside the lowest proportion classified as normal (9.2%). The overall distribution of TBS categories differed significantly across fracture phenotypes (*P < *0.001), as shown in Table 2.

**Table 2:** TBS classification categories by fracture phenotype.

TBS category (mean TBS)	Overall (*n* = 2782, 100%)	No fracture (*n* = 1517, 54.53%)	Single hip fracture (*n* = 156, 5.61%)	Single non-hip fracture (*n* = 763, 27.43%)	Multiple non-hip fractures (*n* = 215, 7.73%)	Multiple fractures incl. hip (*n* = 131, 4.71%)
Normal (≥1.35)	619 (22.25%)	409 (26.96%)	28 (17.95%)	138 (18.09%)	32 (14.88%)	12 (9.16%)
Partially Degraded (1.20–1.35)	1558 (56.00%)	875 (57.68%)	84 (53.85%)	413 (54.13%)	121 (56.28%)	65 (49.62%)
Degraded (≤1.20)	605 (21.75%)	233 (15.36%)	44 (28.21%)	212 (27.79%)	62 (28.84%)	54 (41.22%)

Values: *n* (%) within each fracture phenotype column (overall % based on total N with non-missing mean TBS). TBS classification thresholds for postmenopausal women: normal ≥1.35; partially degraded 1.20–1.35; degraded ≤ 1.20 [9]. Pearson chi-squared test for association between TBS category and fracture phenotype. Results are statistically significant *P* < 0.001.

When looking at the mean TBS between groups, patients with no fractures had the highest mean TBS, indicating better bone microarchitecture (1.29 ± 0.09). Mean TBS values were similar amongst patients with single hip (1.26 ± 0.09), single non-hip (1.26 ± 0.10) and multiple non-hip fractures (1.25 ± 0.09). Interestingly, patients with multiple fractures including at least one hip fracture had the lowest mean TBS (1.22 ± 0.09). One-way ANOVA demonstrated significant differences across fracture phenotypes (F = 34.54, *P* < 0.001), with Tukey’s HSD post-hoc analysis confirming that the multiple fractures including at least hip group differed significantly from all other groups. These results are seen in Table 3.

**Table 3:** Mean TBS compared between the different fracture phenotypes.

Fracture phenotype	N	Mean TBS (SD)	Tukey group
No fracture	1517	1.292 (0.093)	a
Single hip fracture	156	1.258 (0.094)	b
Single non-hip fracture	763	1.259 (0.096)	b
Multiple non-hip fractures	215	1.251 (0.091)	b
Multiple fractures incl. hip	131	1.221 (0.088)	c

Values: mean (SD) of mean TBS (v4). One-way ANOVA: F = 34.54, *P* < 0.001. Tukey’s HSD post-hoc test applied. Tukey group: categories sharing the same letter are not significantly different from each other (*P* > 0.05). Rank: 1 = highest mean TBS (healthiest trabecular microarchitecture).

#### Association between TBS and different fracture phenotype using multinomial logistic regression modelling

In our adjusted multinomial logistic regression model, each 0.1-unit decrease in mean TBS was not associated with single hip fracture (OR 1.01, 95% CI 0.90–1.13, Bonferroni-adjusted *P* = 1), but was significantly associated with single non-hip fracture (OR 1.37, 95% CI 1.28–1.48, Bonferroni-adjusted *P* < 0.001), multiple non-hip fractures (OR 1.32, 95% CI 1.27–1.36, Bonferroni-adjusted *P* < 0.001) and multiple fractures including at least one hip fracture (OR 1.68, 95% CI 1.16–2.43, Bonferroni-adjusted *P* < 0.02) following Bonferroni correction. These findings can be seen in Table 4.

**Table 4:** Adjusted multinomial logistic regression model looking at mean TBS and different fracture phenotypes.

TBS parameter	Single hip vs no fracture	Single non-hip vs no fracture	Multiple non-hip vs no fracture	Multiple incl. hip vs no fracture
Mean TBS (v4)	1.01 (0.90–1.13)	1.37 (1.28–1.48)***	1.32 (1.27–1.36)***	1.68 (1.16–2.43)*

Values: OR (95% CI) per 0.1-unit decrease in TBS (OR >1 = higher fracture risk with lower TBS). Asterisks = Bonferroni-adjusted significance (

* *P* < 0.05,

** *P* < 0.01,

*** *P* < 0.001).

Reference category = no fracture (multinomial logistic regression) for all four columns. Bonferroni correction applied jointly across all four tests in this model family. One adjusted model per TBS parameter.

### Secondary analysis

In our secondary analysis we first evaluated whether the proportion of patients with degraded TBS changed across the two predefined ordered fracture phenotype groups. Within the hip fracture ordered group, there was a significant positive trend in the prevalence of degraded TBS, increasing from 15.69% among patients without hip fracture to 42.75% among patients with multiple fractures including at least one hip fracture (Cochran–Armitage trend test, χ^2^ = 69.16, *P < *0.001). Similarly, within the non-hip fracture ordered groups, the prevalence of degraded TBS increased from 15.69% among patients with no fracture to 28.84% among those with multiple non-hip fractures (Cochran–Armitage trend test, χ^2^ = 48.43, *P < *0.001). These findings can be seen in Table 5.

**Table 5:** Cochran–Armitage trend test looking at rates of degraded bone as per TBS across our two ordered pathways.

Severity level	N	Degraded TBS, *n* (%)
**Hip fracture ordered categories (Cochran–Armitage trend test: χ^2^ = 69.16, df = 1, *P* < 0.001)**		
No fracture	1517	238 (15.69%)
Single hip fracture	156	45 (28.85%)
Multiple fractures incl. hip	131	56 (42.75%)
**Non-hip fracture ordered categories (Cochran–Armitage trend test: χ^2^ = 48.43, df = 1, P < 0.001)**		
No fracture	1517	238 (15.69%)
Single non-hip fracture	763	212 (27.79%)
Multiple non-hip fractures	215	62 (28.84%)

Degraded TBS defined as mean TBS ≤ 1.20. N = patients with non-missing mean TBS at that severity level. The Cochran–Armitage trend test (via prop.trend.test) tests whether the proportion with degraded TBS increases monotonically across the ordered severity levels within each pathway. This is an UNADJUSTED bivariate trend test (no covariate adjustment is possible for this test); the covariate-adjusted association – including left femoral total T-score – is reported separately in Table 6 (ordinal logistic regression).

In the adjusted ordinal logistic regression models, each 0.1-unit decrease in mean TBS was associated with increased odds of being in a more severe hip fracture category (OR 1.34, 95% CI 1.02–1.76, Bonferroni-adjusted *P *= 0.07); however, this association did not remain statistically significant after Bonferroni correction. In contrast, lower mean TBS remained significantly associated with increased odds of being in a more severe non-hip fracture category (OR 1.31, 95% CI 1.16–1.47; Bonferroni-adjusted *P* < 0.001). These results are presented in Table 6.

**Table 6:** Adjusted ordinal logistic regression models looking at mean TBS and hip and non-hip ordered categories.

TBS parameter	Hip pathway OR (95% CI) (Order: no fracture, single hip, multiple incl. hip)	Non-hip pathway OR (95% CI) (Order: no fracture, single non-hip, multiple non-hip)
Mean TBS (v4)	1.34 (1.02–1.76)	1.31 (1.16–1.47)***

Proportional-odds (ordinal logistic regression) models, one per TBS parameter and pathway. OR = odds ratio per 0.1-unit decrease in TBS for being in a more severe fracture category (OR > 1 = higher odds with lower TBS); under the proportional-odds assumption this OR applies uniformly across all thresholds of the ordinal outcome. Each model adjusted for: age at scan, sex (factor), BMI, current glucocorticoid use, rheumatoid arthritis, excess alcohol, current smoking, family history of fracture, left femoral total T-score. Asterisks = Bonferroni-adjusted significance, corrected within this ordinal-regression family only (2 tests) (

* *P* < 0.05,

** *P* < 0.01,

*** *P *< 0.001).

## Discussion

Our study extends the existing literature by examining the association between TBS and a broad spectrum of fragility fracture phenotypes within a large UK clinical cohort. In addition to evaluating individual fracture phenotypes, we investigated whether lower TBS had a graded association with progressively more severe fracture patterns, providing further evidence for the clinical utility of TBS in routine practice.

Exploratory analyses demonstrated that most patients across all fracture phenotypes had partially degraded TBS. Additionally, mean TBS values were also broadly similar across fracture phenotypes, with the exception of patients with multiple fractures including at least one hip fracture, who had significantly lower TBS, although the absolute difference was modest. Despite this, the proportion of patients with degraded TBS increased progressively with fracture severity in both the hip and non-hip ordered groups, a trend confirmed by the Cochran–Armitage trend test. These exploratory analyses suggested that TBS is unlikely to be useful in isolation, but likely to be useful as part of a holistic approach to calculating fracture risk alongside established risk factors such as bone mineral density and FRAX [18]. This interpretation is further supported by our logistic regression models, which demonstrated significant associations between lower TBS and multiple fragility fracture phenotypes after adjustment for relevant clinical risk factors.

Multinomial logistic regression demonstrated that lower TBS was independently associated with single non-hip fractures, multiple non-hip fractures, and multiple fractures including at least one hip fracture. In contrast, the association with isolated hip fracture was not statistically significant. As our single non-hip fracture category largely comprised fractures included within the FRAX major osteoporotic fracture definition, these findings support the association between TBS and major osteoporotic fractures within a UK clinical population, which echoes findings in international cohorts [10]. The absence of an association with isolated hip fracture likely reflects the way fracture phenotypes were defined in our study, as patients with a history of hip fracture who subsequently sustained another fracture were not classified within the isolated hip fracture group. Larger cohorts or studies using conventional hip fracture definitions may therefore be required to clarify this relationship.

When evaluating whether a graded association exists, we first performed a Cochran-Armitage trend test which suggested that more severe fracture phenotypes across hip and non-hip groups had higher rates of patients with degraded TBS. We do acknowledge that our ordered models do not fully capture the complex sequence of fracture events patients experience, though they do provide a pragmatic and clinically interpretable framework for assessing fracture severity within the current study. Our ordinal logistic regression model demonstrated that lower mean TBS was associated with progressively more severe non-hip fracture phenotypes, even after adjustment for potential confounders. A similar association was not observed for the ordered hip fracture models, which we believe is likely attributable to limited statistical power rather than a true absence of association.

To our knowledge, this is the first study to demonstrate a graded relationship between TBS and increasing fragility fracture burden. These findings suggest that TBS could be useful in identifying patients with a higher likelihood of experiencing multiple non-hip fragility fractures. Previous studies have identified established predictors of multiple fragility fractures, including advancing age, prior fracture, falls, lower hip and spine bone mineral density, and rheumatoid arthritis [19]. More recent work has also identified novel predictors, including the combination of alcohol consumption and falls risk [20]. However, it is of interest to note that these studies generally defined multiple fragility fractures as a binary variable [19, 20], whereas our approach captures multiple fragility fractures in a more nuanced approach, providing greater clinical granularity.

The absence of significant associations within the isolated hip fracture and ordered hip fracture models should be interpreted cautiously. Previous studies, including the osteoporotic fractures in men study, have reported that TBS provides limited incremental value for hip fracture prediction beyond established risk factors [21], while meta-analyses have shown only modest improvements in fracture prediction following incorporation of TBS into FRAX [10]. Given the relatively small number of hip fracture events within our cohort and a reported modest/small effect size with hip fractures [10], it may be the case that our study lacked the statistical power to detect this association. It is also plausible that because TBS is derived from lumbar spine DXA images, it may preferentially capture skeletal characteristics relevant to vertebral and other non-hip fracture phenotypes, whereas hip fractures are more strongly influenced by proximal femoral characteristics [22]. Future studies evaluating hip-specific measures of trabecular microarchitecture may clarify whether anatomical site influences fracture prediction, with these measures currently being under development [23]. Given that TBS is derived at the lumbar spine it would have also been useful to investigate vertebral fracture either separately or within our fracture classification; however, we opted against this due to limited sample size. Previous studies have, however, shown TBS to be a strong predictor of lumbar spine fracture risk [24]; hence, further research investigating multiple fracture phenotypes should further consider lumbar fractures using more granular approaches.

It is also important to consider the benefits of identifying patients early who may be at risk of experiencing multiple fragility fractures. Conventional fracture risk tools estimate 10-year fracture probability and primarily guide initiation of antiresorptive therapy [25]. However, a substantial proportion of patients sustain subsequent fragility fractures within the first year following an initial fracture, representing a period of imminent fracture risk [26]. These patients may benefit from more intensive treatment strategies, including anabolic therapies such as teriparatide or romosozumab, or intravenous zoledronic acid where appropriate [25]. Although further validation is required, our study provides preliminary evidence that TBS is associated with a higher likelihood of multiple fragility fractures, and patients with lower TBS in this context may warrant consideration for more intensive intervention.

While our study provides preliminary evidence of a graded association between TBS and increasingly severe fracture phenotypes, the cross-sectional design limits the ability to draw causal conclusions. Hence, it is not clear whether a decrease in TBS occurs prior to a cascade of fractures occurring or whether the reverse is true. Consequently, prospective longitudinal studies are needed to clarify this relationship further, as well as to investigate whether pharmacological interventions can modify this relationship.

## Strengths and limitations

The strengths of our study include a large sample size, adjustment for FRAX risk factors, the use of DXA-derived bone density measurements as well as the utilization of the latest version of TBS.

Limitations include its cross-sectional design within a clinical population and a predominance of female Caucasian participants, which may limit generalizability. Additionally, as patients were referred to the service rather than sampled randomly, there is potential for confounding by indication. Furthermore, given our fracture information was based on patient-reported fracture, this could introduce a source of recall bias. Finally, it is important to appreciate we could not measure TBS on all patients referred to our scanner over the scanners operational period, which may be another source of bias in our study.

## Conclusions

Lower TBS, reflecting degraded trabecular bone microarchitecture, was independently associated with non-hip fragility fractures after adjustment for FRAX variables including BMD in this UK clinical cohort. Lower TBS was also associated with increasing fracture burden across non-hip fracture phenotypes, with ordinal regression demonstrating an independent graded association between lower TBS and more severe ordered non-hip fracture categories. A similar association was not observed for the hip fracture ordered categories. These findings suggest that TBS may provide additional information beyond conventional binary fracture outcomes by demonstrating associations with differing fracture phenotypes and fracture burden. However, prospective longitudinal studies are required to determine whether TBS predicts the development of multiple fragility fractures and to evaluate its potential role in multiple fracture risk stratification in UK and international populations.

## Data Availability

Data can be made available upon reasonable request to the corresponding author.
